# Open stent grafting for complex diseases of the thoracic aorta: clinical utility

**DOI:** 10.1007/s11748-012-0151-y

**Published:** 2012-09-29

**Authors:** Naomichi Uchida

**Affiliations:** Division of Surgery, Graduate School of Biomedical Sciences, Hiroshima University, 1-2-3 Kasumi, Minami-Ku, Hiroshima, 734-8551 Japan

**Keywords:** Open stent grafting, Elephant trunk, Thoracic aneurysm, Aortic dissection, Spinal cord injury

## Abstract

Open stent grafting is an alternative treatment for extensive thoracic aortic replacement. However, this procedure is associated with a high incidence of spinal cord injury, which has limited its application. Multiple factors have been suggested to explain the risk of spinal cord injury, including deep delivery of the stent graft, history of operation of the downstream aorta, and postoperative low blood pressure. Cerebrospinal fluid drainage or a hybrid operation in combination with trans-femoral thoracic stent grafting is useful for preventing spinal cord injury. Open stent grafting remains an alternative treatment for atherosclerotic aneurysms with dilatation of the ascending aorta. Open stent grafting for acute aortic dissection is effective for remodeling of the false lumen. The graft diameter for aortic dissection should be 90 % of the total diameter of the aorta, and the distal landing zone should be limited to the T7 vertebral level to prevent new intimal tears or spinal cord injury. Open stent grafting seems a feasible bailout strategy for the treatment of retrograde aortic dissection after TEVAR for type B aortic dissection. Newly designed devices for open stent grafts include the Matsui-Kitamura stent graft or branched open stent graft, which is produced in Japan. The effectiveness of open stent grafting in the treatment of Marfan syndrome remains unclear. A commercially available device for open stent grafting would be desired in Japan. In conclusion, an open stent graft remains an alternative treatment for complex thoracic aortic pathologies.

## Introduction

The treatment of complex aortic pathologies involving the ascending aorta, aortic arch, and descending aorta are still considered a challenge for many cardiovascular surgeons. Operative methods have been developed to achieve extensive aortic replacement, such as a 1-stage approach, a 2-stage approach, and hybrid treatments using a stent graft. The elephant trunk technique was initially introduced by Borst [1] in 1983 as the first stage toward the development of a 2-stage approach for repairing descending thoracic aortic aneurysms.

The open stent grafting (OSG) technique was first introduced by Kato [2] in 1994 as a method of antegrade replacement of a stent graft into the descending aorta through the open aortic arch under circulatory arrest with a median sternotomy. This technique is associated with several advantages as a combined surgical and interventional approach and therefore appears to be promising. The objective of the current report is to determine the feasibility of this technique for the treatment of different aortic pathologies involving the extensive thoracic aorta. OSG was originally introduced as a method for type B aortic dissection with complete closure of the false lumen. OSG has been used for the repair of distal arch aneurysm and for the exclusion of arteriosclerotic aneurysms, especially in high-risk patients with extensive aortic arch aneurysms in whom distal anastomosis through the median sternotomy approach is difficult. The OSG method was performed extensively between the 1990s and 2000s in Japan. However, a high incidence of spinal cord injury associated with OSG has caused a significant decrease in the number of institutions performing this technique in recent years [3]. Moreover, several alternative treatments have been developed, including the use of commercially available stent grafts on the descending aorta through the trans-femoral artery, the hybrid treatment consisting of a combination of the conventional elephant trunk procedure and thoracic endovascular aortic repair (TEVAR) [4], and minimally invasive surgery with supra-aortic debranching or the chimney method.

On the other hand, in recent years, cases in which the ascending aorta has limited stent graft use, such as dilated ascending aorta or shaggy aorta, OSG has proved to be a safe and less invasive treatment in combination with aortic replacement and debranching with OSG on the upstream aorta and TEVAR on the downstream aorta.

Concerning the stent graft treatment for the closure of primary tears during aortic dissection, TEVAR is currently recommended as first-line treatment for type B aortic dissection, and the prognosis of a downstream aorta is improved by prophylactic stent grafting for type B aortic dissection [5]. In addition, good aortic remodeling can be expected using OSG for acute type A aortic dissection, and the prognosis of a downstream aorta is improved by prophylactic OSG treatment for acute A type aortic dissection.

This paper describes the indications, procedures, applications, and adverse effects such as spinal cord injury or Marfan syndrome that are associated with the use of OSG for different pathologies. In this paper, we have also examined potential alternative techniques for the repair of extended thoracic aortic aneurysm.

## Open stent grafting for atherosclerotic aneurysm of the distal arch

### Indication

Operative indications for OSG were limited to patients considered to have a relatively high operative risk as follows: (1) distal arch aneurysms that could not be treated by thoracic endovascular aortic repair involving the left subclavian artery to the ascending aorta and (2) difficulty in using a median approach extending to the upper middle descending aorta to perform distal anastomosis [6]. Operative indications for OSG included extended thoracic aneurysm in patients with pulmonary complications, repeat surgery, or aging patients in whom it is desirable to avoid left thoracotomy. Recently, the less invasive hybrid TEVAR technique targeting landing zone 0 with supra-aortic debranching or chimney techniques have taken the place of OSG [7, 8]. However, extended thoracic aneurysms requiring replacement instead of stent grafting of the ascending aorta, when the diameter is more than 40 mm, are suitable for OSG. The diameter of a normal distal edge is less than 35 mm on a preoperative computed tomography (CT) scan. If the diameter of the distal edge is more than 35 mm on preoperative CT, a hybrid operation combining OSG with TEVAR is recommended. The stent graft is usually set at 110–120 % of the diameter of the distal descending aorta, and more than 5 cm from the distal normal edge on preoperative 3-dimensional CT scans.

### Procedure

There are 2 types of operations commonly used: supra-aortic bypass to the left subclavian artery and/or the left carotid artery from the ascending aorta and total aortic arch replacement [9]. In both operations, a median sternotomy is performed. Retrograde cerebral perfusion or antegrade selective cerebral perfusion is performed as a preventive measure during OSG placement. The graft is positioned above the aortic valve level, usually between the 7th and 8th thoracic vertebrae (T), and the position of the stent graft is confirmed with real-time trans-esophageal echo guidance. Distal perfusion during open stent deployment and fixation to the native aorta is effective for removing debris and distal protection including renal function. Large occlusion balloon products, i.e., TMP Lock Balloon Catheter L-5 Type (Tokai Medical product, Kasugai, Japan) or Equalizer TM (Boston Scientific, Cork, Ireland) are useful for distal perfusion of the femoral artery in cases of occlusion during stent grafting.

### Early results

Six published series found in PubMed are summarized in Table 1 [3, 6, 10–13]. The average mortality in the analyzed series was 4.7 %. These results are similar to those of open aortic arch repair, and these results are considered acceptable when compared with the 9.5 % mortality rate reported after first-stage repair with the conventional elephant trunk procedure [14–19], 10.0 % mortality rate after 1-stage extensive aortic replacement with left thoracotomy [20–25], or 9.8 % mortality rate after TEVAR to the landing zone 0 with total arch rerouting [26–31] (Table 2).

**Table 1:** Case series and results of open stent grafting for atherosclerotic aneurysm of the distal arch

References	Years	Number	In-hospital mortality	Stroke	Spinal cord injury	Secondary intervention on the downstream aorta
Usui [3]	2002	24	0	1 (4 %)	4 (17 %)	
Sueda [10]	2004	34	2 (5.9 %)	1 (2.9 %)	1 (2.9 %)	1 (2.9 %)
Flores [11]	2006	25	3 (12 %)	4 (16 %)	6 (24 %)	
Shimamura [12]	2008	69	2 (2.9 %)		4 (5.8 %)	4 (5.8 %)
Uchida [6]	2010	58	1 (1.7 %)	2 (3.4 %)	2 (3.4 %)	1 (2 %)
Di Bartolomeo [13]	2010	22	3 (14 %)	7.50 %	2 (8.1 %)	3 (14.9 %)
		210	11 (4.7 %)		19 (8.2 %)	9 (4.3 %)

**Table 2:** Case series and results of other procedures among conventional elephant trunk procedure, 1-stage extensive aortic replacement, and endovascular stent grafting to the landing zone 0 with total arch rerouting

Procedure	References	Years	Number	In-hospital mortality	Stroke	Spinal cord injury
Conventional elephant trunk	Svensson [14]	2004	94	7 (18.4 %)	5 (5.3 %)	2 (2.1 %)
	Coselli [15]	2005	38	7 (18.4 %)	2 (5.3 %)	1 (3 %)
	LeMaire [16]	2006	148	21 (14.1 %)	9 (6.1 %)	2 (1.3 %)
	Safi [17]	2007	254	27 (10.6 %)	5 (2 %)	1 (0.4 %)
	Etz [18]	2008	215	16 (8 %)	14 (6.5 %)	2 (0.9 %)
	Toda [19]	2009	111	4 (3.6 %)	2 (1.8 %)	9 (8.1 %)
	Total		860	82 (9.5 %)	37 (4.3 %)	17 (2.0 %)
One-stage approach	Minale [20]	1994	12	2 (16 %)	0	0
	Massimo [21]	1997	34	5 (14.7 %)	0	3 (8.8 %)
	Beaver [22]	2001	14	2 (14 %)	1 (7 %)	2 (14 %)
	Doss [23]	2007	15	1 (6.6 %)	3 (20 %)	0
	Kouchoukos [24]	2007	69	5 (7.2 %)	0	1 (1.4 %)
	Okada [25]	2009	16	1 (6.3 %)	1 (6 %)	1 (6.3 %)
	Total		160	16 (10 %)	5 (3.1 %)	7 (4.4 %)
Debranching of the aortic arch and TEVAR	Melissano [26]	2007	64	4 (6.3 %)	2 (3.1 %)	2 (3.1 %)
	Gottardi [27]	2008	73	5 (6.8 %)	1 (1.4 %)	0
	Kurimoto [28]	2009	104	8 (7.7 %)	5 (4.9 %)	3 (2.9 %)
	Kotelis [29]	2009	88	17 (19 %)	5 (4.9 %)	1 (1.1 %)
	Hughes [30]	2009	28	0	0	1 (3.6 %)
	Donas [31]	2010	20	3 (15 %)	3 (15 %)	0
	Total		377	37 (9.8 %)	16 (4.2 %)	7 (1.9 %)

### Spinal cord injury

The rate of incidence of paraplegia after OSG was reported to be 8.2 %. Flores et al. [11] reported that in 6 (24 %) patients with spinal cord injury, a significant difference was observed in the mean value of the thoracic vertebral level. Spinal cord injury after OSG was associated with several factors such as the thoracic vertebral level where the distal end of the OSG was deployed, a history of downstream aortic surgery, the simple clamping of the left subclavian artery without perfusion, intraoperative hypotension, and individual pathologic condition (aneurysm or acute dissection) of each patient. Cerebrospinal fluid drainage was performed before the operation in elective cases with a history of aortic repair in the thoracoabdominal or abdominal aorta or when the pathologic condition required stent graft delivery lower than T9. Shimamura et al. [12] performed cerebrospinal fluid drainage before the operation in patients with a history of repair of the downstream aorta and when the pathologic condition required stent graft delivery lower than T9. In these cases, a short stent graft was delivered in the descending aorta without excluding the aneurysm, and additional endovascular grafting was performed the next day.

### Re-intervention

The average rate of secondary intervention on the downstream aorta after OSG is 4.3 % [3, 6, 10–13]. In these series, the patients who underwent scheduled 2-stage TEVAR were included, and the addition of second-stage TEVAR rather than placement of long OSG appears to be beneficial for the prevention of spinal cord injury. The use of the elephant trunk technique with or without a stent remains controversial. Elephant trunk without a stent as a floppy graft within an aneurysm is associated with the risk of dilated aneurysm or technical difficulty requiring the insertion of a guide wire at the second-stage surgery or the risk of generating a type III endoleak between the elephant trunk and TEVAR. Interval mortality between the first and second stage, during the wait for the second operation, ranged from 12 to 25 % [14–19].

### Hybrid procedure

A hybrid repair consisting of a first-stage proximal reconstruction using either a conventional arch elephant trunk technique or the “stented” elephant trunk technique (open stent grafting) and a second-stage endovascular repair of the descending thoracic aorta using OSG as the proximal landing zone has been described. This technique is reserved for patients presenting with extensive thoracic aortic aneurysms involving the ascending, transverse arch and descending thoracic aorta, or the so-called mega aorta syndrome. Because of these hybrid arch reconstructive techniques, surgeons can extend the indications and provide an alternative surgical option for patients who were previously considered too high-risk for conventional open repair of aortic arch aneurysms.

## Prophylactic open stent grafting for type A aortic dissection

### Effectiveness of the OSG for aortic dissection

The effectiveness of OSG for distal stent grafting combined with proximal arch replacement has been reported. The aim of this technique is not only to repair the intimal tear of the proximal descending aorta in the so-called “retrograde type A aortic dissection” using a stent graft, but also to exclude all antegrade blood flow in the false lumen of the thoracic aorta while re-expanding the true lumen and obliterating the false lumen in the descending aorta. This serves to restore distal visceral and limb perfusion and prevent aortic dilatation after surgery. The reported incidence of patency and blood flow within the false lumen after conventional surgical repair ranges from 31 to 89 % (Table 3) [32–39]. A persistent patent false lumen in the downstream aorta exposes the patient to malperfusion syndrome and aortic aneurysmal dilatation. On the other hand, the reported incidence of patent false lumen on the proximal descending aorta after hybrid open stent grafting decreases to less than 7 %, and the distal reoperation rate is less than 6 % when endovascular re-intervention is performed (Table 4) [12, 43–46]. After the OSG, the false lumen enlarged the true lumen, reestablished flow in the true lumen and the side-branches, approximated the 2 dissected layers, promoted thrombosis of the distal residual dissected aorta, helped to remodel the dissected aortic wall, or contributed to shrinkage of the aorta. Because of encouraging surgical results obtained, this procedure may become the new “standard” therapy for type A dissection involving the repair of the aortic arch. However, patients with type A dissection are not eligible for randomization because of unstable hemodynamics, the need for emergency surgery, a broad spectrum of clinical conditions, and different extents of the proximal and distal propagation and involvement of aortic dissection.

**Table 3:** Series comparisons between conventional procedures without open stent grafting (non OSG) and open stent grafting (OSG) procedures

References	Years	Procedure	Number	Early mortality (%)	Mean follow-up (months)	Patent false lumen (%)	Distal reoperation (%)
Kirsch [32]	2002	Non-OSG	160	32.50	54		23
Kazui [33]	2002	Non-OSG	240	13.80			22.80
Halstead [34]	2007	Non-OSG	179	13.40	61	43	16
Geirsson [35]	2007	Non-OSG	221	12.70	49		23.40
Kimura [36]	2008	Non-OSG	218	7.30	56	64	12
Jakob [37]	2008	Non-OSG	23	21.70	48	89.00	33.00
Fattouch [38]	2009	Non-OSG	189	15.60	88	31.00	36.20
Park [39]	2009	Non-OSG	122	5.10	34	81.10	15.60
Total		Non-OSG	1352	14.30		54	21.30
Shimamura [12]	2008	OSG	41	4.90	60		0
Sun [40]	2009	OSG	107	3.70	35	5	0
Desai [41]	2009	OSG	36	14		<20	0
Uchida [42]	2010	OSG	66	4.50	74	3	3
Tsagakis [43]	2010	OSG	106	12	20	7	6
Total		OSG	356	7.60		5	2.50

**Table 4:** Case series and results of open stent grafting for type A aortic dissection

References	Years	Number (acute/chronic)	In-hospital mortality	Stroke	Spinal cord injury	Secondary intervention on the downstream aorta
Shimamura [12]	2008	57 (31/26)	2 (3.5 %)	Unknown	4 (7 %)	0
Tsagakis [43]	2010	68 (68/0)	9 (13 %)	7 (10 %)	1 (1 %)	7 (10 %)
Uchida [44]	2011	80 (80/0)	4 (5.0 %)	2 (2.5 %)	0	1 (1.5 %)
Sun [40]	2011	291 (146/145)	9 (3.1 %)	7 (2.4 %)	7 (2.4 %)	1 (0.3 %)
Pacini [45]	2011	90 (20/70)	11 (12 %)	1 (1.1 %)	8 (9 %)	18 (20 %)
Hoffman [46]	2012	32 (32/0)	1 (3.1 %)	0	0	0
Total		618 (377/241)	36 (5.8 %)		20 (3.2 %)	27 (4.4 %)

### Indication

The primary tear in type A aortic dissection (TAAD) is located on the ascending aorta in 70 % of all cases, the aortic arch in 10 %, and the descending aorta in 20 % [48]. Thus, there is retrograde type A aortic dissection in 20 % of all cases where the primary tear remains after standard repair for TAAD. Residual false lumen on the distal aorta without resection of the primary tear not only requires distal reoperation, but also induces visceral and/or limb malperfusion. In the early postoperative period, several patients treated for acute TAAD experienced intestinal necrosis caused by poor perfusion of intra-abdominal branches of the aorta, and such malperfusion often occurs because the distal false lumen has remained patent with the collapsed true lumen. The OSG using a stent graft can exclude antegrade blood flow in the false lumen of the thoracic aorta, thus restoring distal visceral and limb perfusion more effectively. The OSG is indicated for re-expansion of the true lumen in cases of occlusion or severe collapse, even if the site of the primary tear is proximally located from the aortic arch. TADD can be performed with arch dilatation or distal aneurysmal change. Fattouch [38] reported a descending aortic diameter of 4.5 cm or larger as a predictor for distal reoperation. Despite the use of aggressive repair techniques with resection of the primary tear, the reported incidence of patent false lumen and distal reoperation remains high. Anastomotic leakage or small tears in the proximal descending thoracic aorta after graft replacement of the ascending aorta or aortic arch increase the susceptibility of the false lumen to dilation because of shear stress acting on the proximal descending thoracic aorta, even if the primary intimal tear is located in the ascending aorta. Distal reoperation is often required for dilatation of a false lumen in the distal descending aorta during the late postoperative period. In addition, the age of patients is a more important predictor of distal aortic reoperation than patent false lumen. Due to the higher need for reoperation in younger patients, a prophylactic arch repair is reasonable considering the long-term prognosis over 10 years after distal repair. The indications for an OSG in type A aortic dissection considering the long-term risk of malperfusion in early-term and aortic events on the downstream aorta are as follows:

1. The primary tear is distally located from the aortic arch (“retrograde dissection”).
2. The true lumen on the distal aorta is occluded or severely collapsed.
3. Arch dilatation is 4.0 cm or larger including chronic type A aortic dissection with the dilation on the distal arch.
4. Younger patients (<70 years of age) with DeBakey type I aortic dissection.

### Procedures

The OSG procedures for aortic dissection are the same as those used for the arteriosclerotic aneurysm repair. However, several procedures for the repair of proximal aortic aneurysm have been reported. Total arch replacement using 4 branch grafts similar to a non-stented elephant trunk method is commonly performed in Japan. Hemiarch replacement using a straight graft instead of total arch replacement has been reported; however, it is associated with an increase in the incidence of residual false lumen compared with total arch replacement.

### Graft selection

Hoffman [46] reported that at midterm follow-up, choosing a stent the size of the total aortic diameter (true and false lumen) allows immediate closure of the false lumen of the aortic dissection, ensuring obliteration of all intercostal arteries without any signs of endoleak or residual patency of the false lumen. Karck [47] have refrained from using stents the size of the total aortic diameter because of the risk of aortic wall damage. We have performed intraoperative direct measurement using a ball-shaped sizer under TEE guidance. A stent graft 10 % smaller than the total aortic diameter was selected, and no new intimal tears occurred in our series.

Another controversial issue is the choice of a distal landing zone for the stent graft. Most surgeons prefer a higher distal landing zone because of the fear of spinal cord injury. Uchida [44] reported the absence of patients with acute type A dissection exceeding level T7. Hoffman [46] decided to stent the aorta down to the T10–T12 vertebral level. Because most patients have a tear within the T7 vertebral level, a deep insertion at the OSG site is not recommended.

### Early results

Table 4 [12, 43–46] summarizes the results of 6 published series on the use of OSG for type A aortic dissection found in PubMed. The average mortality in the series analyzed was 5.8 %. This is considered acceptable in comparison to the mortality of 8.9 % reported after tear-oriented conventional aortic replacement. When endovascular re-intervention is easily performed, the reported incidence of patent false lumen in the proximal descending aorta after hybrid OSG surgery decreases to <7 %, with the distal reoperation rate at <6 %.

### Spinal cord injury

The reported rate of spinal cord injury after OSG is 3.2 % [12, 43–46]. However, most cases of spinal cord injury are associated with chronic aortic dissection [12, 40, 45]. The OSG is not recommended when the lower extremity or visceral organs are perfused through the false lumen or when the false lumen contains substantial thrombus in cases of chronic aortic dissection. Arteriosclerotic aneurysm is associated with a high risk of atheromatous embolism or disorder of the collateral circulation network because of arteriosclerotic changes of the downstream aorta including distal landing aorta compared with aortic dissection. Although chronic aortic dissection has a lower risk of atheromatous embolism, direct circulation to the anterior spinal artery via intercostal arteries from the false lumen can be affected by closure of entry even when the FET is not deployed extensively. On the other hand, when the intercostal arteries are dissected during acute aortic dissection, direct intercostal perfusion remains through the false lumen because a secondary tear exists between the true and false lumen. When an acute dissection with a thrombosed false lumen expands behind the descending aorta, spinal cord ischemia can occur before the operation because of lack of communication between the true and false lumens. This communication remains on the downstream aorta after deployment of the OSG in patent false lumen. Therefore, the risk of spinal cord injury is low after OSG for acute aortic dissection. Two patients in our series who had spinal cord ischemia before the emergency operation had a thrombosis of the false lumen that widely expanded behind the descending aorta according to preoperative CT. These patients remained paraplegic after the operation despite the maintenance of high mean pressure after the operation.

## Entry closure using the OSG for type B Aortic dissection

### Indications

Concerning the stent graft treatment for the closure of primary tears during aortic dissection, TEVAR is currently recommended as first-line treatment for complicated type B aortic dissection with malperfusion or dilatation [5]. The effectiveness of the entry closure is not clear for chronic type B dissection. Operative strategies for dilatation in the aortic arch, the ascending aorta, or the aortic route with type B aortic dissection are controversial. The OSG with upstream aortic replacement has been reported as an alternative treatment. TEVAR for type B aortic dissection with primary entry near the left subclavian artery origin is associated with anatomical difficulties in managing the arch vessels using special materials such as fenestrated grafts or branch grafts. Furthermore, the discrepancy between the proximal diameter with an undissected aorta and the distal diameter with a collapsed true lumen sometimes requires a stent graft of the tapering type. The OSG allows effective management of arch vessels with abundant atherosclerotic plaque, dilatation of the ascending aorta, and annuloaortic ectasia [49]. However, TEVAR might be able to achieve better results in difficult cases in the future with the progress in stent-graft materials.

### Bailout strategy of retrograde dissection after TEVAR

Retrograde type A aortic dissection after TEVAR was reported in 3 % (2/66) of patients in a continental registry [50]. Gorlitzer [51] reported that retrograde aortic dissection occurred in 4 of 29 patients (13.8 %) undergoing TEVAR for acute complicated aortic type B dissection. The use of OSG seems a feasible bailout strategy for the treatment of retrograde aortic dissection after TEVAR.

## Marfan syndrome

The effectiveness of OSG in the treatment of Marfan syndrome remains unclear. Sun [52] concluded that OSG for type A aortic dissection (acute 19, chronic 25) was a suitable alternative in patients with Marfan syndrome. However, further research is necessary, in particular in acute dissection in patients with Marfan syndrome with a fragile intima.

## Open stent grafting devices

Since the OSG technique was first introduced by Kato [2] in 1994, homemade stent grafts have been used in Japan. A synthetic graft was attached by means of a self-expandable Z-shaped stent (William Cook Europe A/S) on the distal side as reported by Kato and colleagues [2]. A Z-shaped or Matsui-Kitamura (MK) stent was used as a self-expandable stent. Because the Z-shaped stent has a risk of perforation in synthetic grafts, the use of 2 stents has been recently recommended. The MK stent is flexible, but cannot be attached to a synthetic graft [53]. Therefore, the use of commercially available open stent grafts is preferable. In Europe, the E-vita-open system is commercially available [54]. Recently, the branched OSG technique has been reported with good results in Osaka University [55], and triple branched OSG for aortic dissection has been performed in Fujian Medical University in China [56]. Open stent grafting with a 4-branched graft has been reported with good results in Hanover [57].

## Conclusion

The use of open stent grafting for extended atherosclerotic aneurysms in high-risk patients is limited because of its invasive nature and its association with a high incidence of spinal cord injury as compared with TEVAR. However, the combination of OSG and TEVAR for complex pathologies of the ascending aorta is effective as an alternative treatment for extensive aortic replacement. OSG may become the new “standard” therapy for type A dissection involving repair of the aortic arch. OSG seems a feasible bailout strategy for the treatment of retrograde aortic dissection after undergoing TEVAR for type B aortic dissection.
